# Genomic and Transcriptomic Insights into Salinity Tolerance-Based Niche Differentiation of *Synechococcus* Clades in Estuarine and Coastal Waters

**DOI:** 10.1128/msystems.01106-22

**Published:** 2023-01-09

**Authors:** Xiaomin Xia, Ying Liao, Jiaxing Liu, Sze Ki Leung, Pui Yin Lee, Lingshuai Zhang, Yehui Tan, Hongbin Liu

**Affiliations:** a Key Laboratory of Tropical Marine Bio-resources and Ecology, South China Sea Institute of Oceanology, Chinese Academy of Sciences, Guangzhou, China; b Guangdong Provincial Key Laboratory of Applied Marine Biology, South China Sea Institute of Oceanology, Chinese Academy of Sciences, Guangzhou, China; c Department of Ocean Science, The Hong Kong University of Science and Technology, Hong Kong, China; d Hong Kong Branch of Southern Marine Science & Engineering Guangdong Laboratory, The Hong Kong University of Science and Technology, Hong Kong, China; Univ. California Merced

**Keywords:** Cyanobacteria, euryhaline *Synechococcus*, salinity, transcriptome, niche partitioning

## Abstract

Cluster 5 *Synechococcus* is one of the most important primary producers on earth. However, ecotypes of this genus exhibit complex geographical distributions, and the genetic basis of niche partitioning is still not fully understood. Here, we report distinct distributions of subcluster 5.1 (SC5.1) and subcluster 5.2 (SC5.2) *Synechococcus* in estuarine waters, and we reveal that salinity is the main factor determining their distribution. Clade III (belonging to SC5.1) and CB4 (belonging to SC5.2) are dominant clades in the study region, with different ecological distributions. We further conducted physiological, genomic, and transcriptomic studies of *Synechococcus* strains YX04-3 and HK05, which are affiliated with clade III and CB4, respectively. Laboratory tests showed that HK05 could grow at low salinity (13 ppt), whereas the growth of YX04-3 was suppressed when salinity decreased to 13 ppt. Genomic and transcriptomic analysis suggested that euryhaline clade CB4 is capable of dealing with a sudden drop of salinity by releasing compatible solutes through mechanosensitive channels that are coded by the *mscL* gene, decreasing biosynthesis of organic osmolytes, and increasing expression of heat shock proteins and high light-inducible proteins to protect photosystem. Furthermore, CB4 strain HK05 exhibited a higher growth rate when growing at low salinity than at high salinity. This is likely achieved by reducing its biosynthesis of organic osmolyte activity and increasing its photosynthetic activity at low salinity, which allowed it to enhance the assimilation of inorganic carbon and nitrogen. Together, these results provide new insights regarding the ecological distribution of SC5.2 and SC5.1 ecotypes and their underlying molecular mechanisms.

**IMPORTANCE** *Synechococcus* is a group of unicellular Cyanobacteria that are widely distributed in global aquatic ecosystems. Salinity is a factor that affects the distribution of microorganisms in estuarine and coastal environments. In this study, we studied the distribution pattern of *Synechococcus* community along the salinity gradient in a subtropical estuary. By using omic methods, we unveiled genetic traits that determine the niche partitioning of euryhaline and strictly marine *Synechococcus*. We also explored the strategies employed by euryhaline *Synechococcus* to cope with a sudden drop of salinity, and revealed possible mechanisms for the higher growth rate of euryhaline *Synechococcus* in low salinity conditions. This study provides new insight into the genetic basis of niche partitioning of *Synechococcus* clades.

## INTRODUCTION

*Synechococcus* (spherical to rod-shaped, 0.6–2.1 μm in diameter) are one of the most abundant and widely distributed photosynthetic organisms on earth (1, 2). They contribute significantly to global primary production. *Synechococcus* species are divided into 5 clusters by their morphological, physiological, and genetic properties (2). Cluster 5 is composed of *Synechococcus*/*Cyanobium* strains that are often found in marine and brackish environments (2). Cluster 5 *Synechococcus* strains can be classified into 3 pigment types (phycocyanobilin-rich [PCB-rich], phycoerythrobilin-rich [PEB-rich], and phycourobilin-rich [PUB-rich]), and 3 phylogenetic subclusters (subcluster 5.1, 5.2, and 5.3), according to the composition of their antenna protein and gene markers (e.g., *petB* and *rpoC1*), respectively (3–6). Phylogenetic subcluster 5.1 (hereafter SC5.1), which is mainly composed of PEB-containing (including PEB-rich and PUB-rich) *Synechococcus*, is further divided into more than 20 phylogenetic clades (3, 5, 7). The global distribution pattern of SCS.1 clades has been reported in previous studies (3, 8–11). Compared with SC5.1, fewer studies have been conducted on subcluster 5.2 (hereafter SC5.2), although it is abundant in estuarine waters, brackish waters, and freshwaters (12–17), as it can cope with variations in salinity (18, 19). Despite SC5.2 being ecologically important in various aquatic systems (20, 21) and being a genetically diverse group (12), clades within SC5.2 still have not been well defined. Finally, subcluster 5.3 (hereafter SC5.3) has been reported in various marine environments and freshwater environments (16), while it has a relatively low abundance in the global ocean (3).

Spatio-temporal niche partitioning of different *Synechococcus* pigment types and phylogenetic clades has been widely observed in estuarine waters or brackish waters along the salinity gradient (13, 22–25). For example, a clear niche partitioning of *Synechococcus* ecotypes has been found in the Pearl River estuary (described as being a salt wedge), such that the dominant *Synechococcus* were shown to shift from freshwater *Synechococcus* ecotypes to PCB-rich SC5.2 clades, and then to PEB-containing SC5.1 clades along the salinity gradient (14, 25). These studies suggest that niche partitioning between SC5.2 PCB-rich *Synechococcus* and SC5.1 PEB-containing *Synechococcus* can be attributed to the fact that these 2 groups have different abilities to cope with variations in salinity as well as distinct light absorptive properties (21, 26). However, there is still not much known about the underlying molecular mechanisms related to niche separation between SC5.2 PCB-rich and SC5.1 PEB-containing *Synechococcus* in estuarine waters.

A combination of gene gains or losses, and sequence divergence are thought to be responsible for the niche separation of *Synechococcus* ecotypes (27). For example, a genome comparison indicated that clade CRD1 has a larger collection of Fe-related genes than the other ecotypes, which helps it survive and succeed in low-iron habitats (27, 28). It has also been reported that heterotrophy genes, such as *bzt* and *potE* (polar amino acid permease genes), are important for the survival of *Synechococcus* in harsh mesopelagic environments (29). In addition, using a combination of genomic and transcriptomic analysis, we previously revealed that the *glzT* gene, which encodes a channel protein with a glycine zipper, is likely to be involved in the low salinity adaptation of euryhaline PEB-containing *Synechococcus* (18). So far, more than 50 complete whole-genome sequences of marine, euryhaline and freshwater *Synechococcu*s are available in the NCBI database or Cyanorak (http://application.sb-roscoff.fr/cyanorak/organisms.html?execution=e2s1) (30). However, most of these genomes are from SC5.1 *Synechococcus* strains. Genome and transcriptome comparisons between SC5.1 PEB-containing strictly marine *Synechococcus* and SC5.2 PCB-rich euryhaline *Synechococcus* have seldom been conducted.

The Pearl River is one of the largest rivers in China, with an average annual discharge of 3.36 × 10^11^ m^3^ (31). The constant mixing between large amounts of freshwater discharge (from the Pearl River) and seawater (from the South China Sea) in summer results in a complex topographic and hydrodynamic environment. Organisms in the Pearl River Estuary also experience strong salinity variation due to the mixing of different water masses modulated by the tidal cycle (32). *Synechococcus* is reported to have a high abundance (up to 7 × 10^5^ cells/mL) and diversity (both phylogenetic and phenotypic) in the Pearl River estuary (19, 33). Hence, this is an ideal location to study the relationship between the *Synechococcus* community and environmental factors.

Here, we investigated the spatial changes in abundance and community structure of *Synechococcus* in the Pearl River estuary using flow cytometry and high-throughput sequencing technology. Moreover, we identified the key factors that determine *Synechococcus* community composition. Finally, and most importantly, we analyzed the genome and transcriptome of 2 isolates representing the dominant *Synechococcus* clades in the Pearl River estuary, and revealed the mechanisms involved in specific niche adaptation of euryhaline *Synechococcus*.

## RESULTS AND DISCUSSION

### Abundance and phylogenetic composition of *Synechococcus* along the salinity gradient.

**FIG 1:**
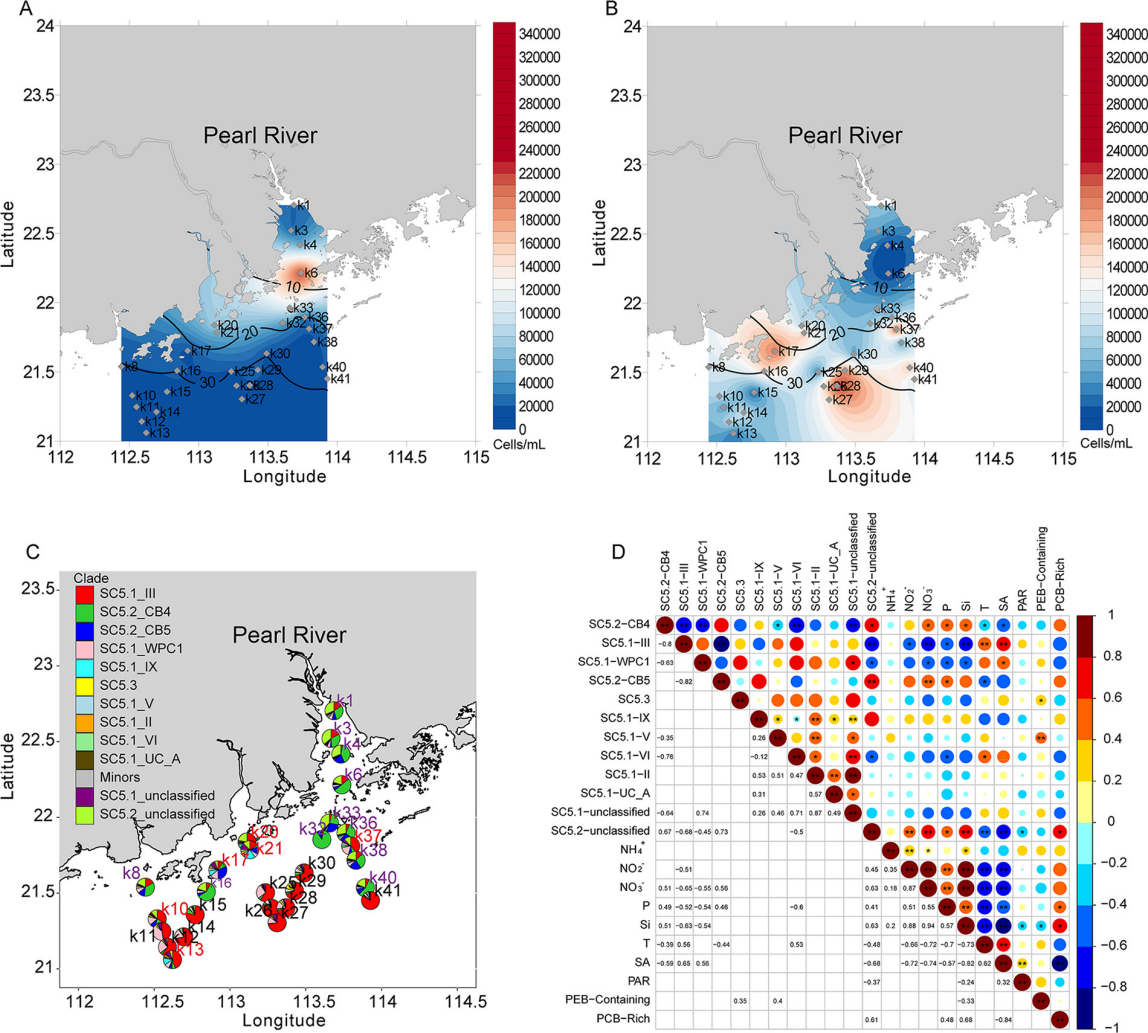
Abundance and community composition of *Synechococcus* in the Pearl River estuary during July 2018. (A and B) Abundance of PCB-rich and PEB-containing *Synechococcus*, respectively. The abundances of *Synechococcus* cells were evaluated using flow cytometry. The contour lines indicate the different salinities of the surface water. (C) *Synechococcus* assemblage compositions. Stations in purple were from the ES biome (Estuarine water dominated), those in red were from the Trans biome (Transitional area), and those in black were from the MA biome (Marine water dominated). Biomes were determined by NMDS analysis of *Synechococcus* community compositions and salinity of sampling stations (Fig. S1). (D) Correlation analysis of the relationship between *Synechococcus* lineages and environmental parameters in the Pearl River estuary. P, phosphorus; T, temperature; SA, salinity. Fig. 1A and 1B were generated using Surfer V15. Fig. 1C and 1D were generated using R.

Along the Pearl River estuary-coastal sea transect, the salinity of the surface water increased gradually from 0.1 ppt to 33.8 ppt (Fig. 1). Both PEB-containing and PCB-rich *Synechococcus* were distributed in our study region, with the abundance of the former ranging from 1.00 × 10^4^ to 2.25 × 10^5^ cells mL^−1^ and that of the latter ranging from 2.00 × 10^3^ to 3.22 × 10^5^ cells mL^−1^ (Fig. 1A andB). These 2 types of *Synechococcus* displayed different distribution patterns in the Pearl River estuary, such that the PEB-containing *Synechococcus* were relatively more abundant in the high salinity waters (> 20 ppt), whereas the PCB-rich *Synechococcus* were generally more abundant in the low salinity waters (0.1 ppt to 20.0 ppt) (Fig. 1A and B). This distribution pattern is consistent with several previous studies (14, 21, 25, 34, 35). It has been suggested that light is one of the major factors determining the distribution of PCB-rich and PEB-containing *Synechococcus* in estuarine waters (20, 22, 36, 37), due to their different light preferences (PEB and PCB have absorption peaks at 550–570 nm and 630 nm, respectively) (4, 21). However, photosynthetically active radiation (PAR) was not a significant factor in determining the spatial distribution pattern of *Synechococcus* ecotypes in the Pearl River estuary (Fig. 1D). Instead, the abundance of PCB-rich *Synechococcus* was significantly positively correlated with phosphate, and negatively correlated with salinity (Fig. 1D). These results suggest that in the Pearl River estuary, nutrient availability (especially phosphate concentration) and salinity might be the main factors in shaping the spatial distribution pattern of *Synechococcus* pigment types.

10.1128/msystems.01106-22.1 FIG S1 (A), NMDS analysis of the *Synechococcus* assemblages in the Pearl River estuary. Three biomes: MA (Marine water dominated), ES (Estuarine water dominated), and Trans (Transitional area) were defined. (B) Mental test analysis of the relationship between the *Synechococcus* phylogenetic compositions in the three biomes and environmental parameters. SA, salinity; PAR, photosynthetically active radiation; T, temperature. P, Phosphate. *, factors significantly correlated with variation in the *Synechococcus* community (*P* < 0.05). Download FIG S1, DOCX file, 0.2 MB. Copyright © 2023 Xia et al. 2023 Xia et al. https://creativecommons.org/licenses/by/4.0/ This content is distributed under the terms of the Creative Commons Attribution 4.0 International license.

In our sampling region, the *Synechococcus* phylogenetic compositions displayed clear spatial variations along the salinity gradient (Fig. 1C). SC5.1 clades I, IV, PAC1, UC-A, XV, and XX were rare in the Pearl River estuary in the summer. This is not surprising because they are known to prefer cold waters or oligotrophic environments (3, 7, 8). The Pearl River estuary could be divided into 3 biomes: MA (Marine water dominated), ES (Estuarine water dominated), and Trans (Transitional area) according to *Synechococcus* community compositions and salinity of sampling stations (Fig. S1A). Salinity was a major factor influencing the niche partitioning of *Synechococcus* clades in the Pearl River estuary (Mantel test; *P* = 0.001; *r* = 0.36) (Fig. S1B). The MA biome was dominated by SC5.1 clades whereas the ES biome was dominated by SC5.2 clades (Fig. 1C). The majority of SC5.2 *Synechococcus* in the study area were affiliated with clade SC5.2-CB4, which was represented by CB0101 (a strain isolated from Chesapeake Bay) and HK05 (a strain isolated from the Pearl River estuary) (Fig. S2). The highest relative abundance of SC5.2-CB4 (accounting for up to 88.8% of the total reads), was detected at sampling station K32, where the salinity was 17.0 ppt. Besides SC5.2-CB4, SC5.2-CB5 was also widely detected in the ES biome, with relative abundance ranging between 3.2 and 25.0%. This demonstrated that in estuarine waters, PCB-rich *Synechococcus* mainly consisted of clades SC5.2-CB4 and SC5.2-CB5. These 2 clades are also abundant in the Chesapeake Bay (12) and were isolated from the Jiulong River estuary (Xiamen, China, strains XM01 and XM24 in fig. S2), suggesting they may be globally distributed in estuarine waters. Although our phylogenetic analysis suggested that SC5.2 may also contain clades formed by *Cyanobium* and *Synechococcus* strains isolated from various environments (e.g., cold oligotrophic waters, freshwater environments, and brackish waters) (Fig. S2 and Data set S2), these clades were rare in the Pearl River estuary waters. Thus, it would be interesting to compare the genome sequences and physiological properties of different SC5.2 ecotypes along the extreme gradient of salinity to help us further understand why they have such distinct geographic distributions.

10.1128/msystems.01106-22.2 FIG S2 Phylogenetic analysis of *Synechococcus*/*Prochlorococcus* strains based on 43 concatenated phylogenetically informative marker genes (A) and *rpoC1* gene (B). The numbers in each node indicate bootstrap values. Clades of SC5.1 (light blue bars) and SC5.2 (dark green bars) were assigned according to previous studies (5, 8). The HK05 and YX04-3 genomes compared in this study are labeled with green and red circles, respectively. The accession numbers of the sequences used in this figure are listed in Data set S2. Download FIG S2, DOCX file, 0.6 MB. Copyright © 2023 Xia et al. 2023 Xia et al. https://creativecommons.org/licenses/by/4.0/ This content is distributed under the terms of the Creative Commons Attribution 4.0 International license.

10.1128/msystems.01106-22.7 DATA SET S2 An Excel file contains information about (i) the origin of SC5.2 strains and their accession numbers; (ii) strains used for phylogenetic analysis; (iii) genome_properties of HK05 and YX04; (iv) annotations of HK05 and YX04. Download Data Set S2, XLSX file, 1.4 MB. Copyright © 2023 Xia et al. 2023 Xia et al. https://creativecommons.org/licenses/by/4.0/ This content is distributed under the terms of the Creative Commons Attribution 4.0 International license.

In the Pearl River estuary, SC5.1 *Synechococcus* were mainly composed of clades III and WPC1, and they were the dominant lineages in the MA biome (Fig. 1C). Correlation analysis demonstrated that these 2 clades were strongly positively correlated with clade VI, but were strongly negatively correlated with SC5.2-CB4 and SC5.2-CB5 (Fig. 1D). This indicates clade III has distinct niche preferences from SC5.2 lineages. Clade III occurs widely in phosphorus-depleted waters (3, 38), since its strains have a particularly high number of phosphatase genes, allowing them to utilize diverse sources of organic phosphorus and adapt to phosphate-depleted environments (39, 40). This might explain why clade III is more abundant than clade II (another warm water dominant clade) in the northern part of the South China Sea in summer, which is a known P-limited environment (41). Correlation analysis showed that SC5.1-clade III was strongly positively correlated with salinity, suggesting its low ability to cope with the stress of low salinity.

### Genome-wide comparison revealing niche partitioning of SC5.1-clade III and SC5.2-CB4 *Synechococcus* representative strains.

SC5.1-clade III and SC5.2-CB4 were the dominant *Synechococcus* ecotypes in the MA and ES biomes, respectively (Fig. 1C), and they both had distinct niche preferences. To unveil the genetic basis of niche partitioning of these 2 clades, representative strains of each, YX04-3 (SC5.1 clade III) and HK05 (SC5.2-CB4), were isolated (Fig. S2). We incubated these 2 strains in f/2 medium at 2 salinities, 13 ppt or 32 ppt, and found that HK05 could grow at both salinities but displayed a higher growth rate and photosynthesis efficiency at 13 ppt, whereas YX04-3 could not survive at a salinity of 13 ppt (Fig. S3). These results further demonstrated that SC5.1 clade III and SC5.2-CB4 *Synechococcus* have different abilities to cope with variations in salinity. HK05 displayed a higher OD640 nm/OD440 nm value at a salinity of 13 ppt, suggesting that it might contain a relatively higher amount of PCB pigment at this lower salinity level (Fig. S3D).

**FIG 2:**
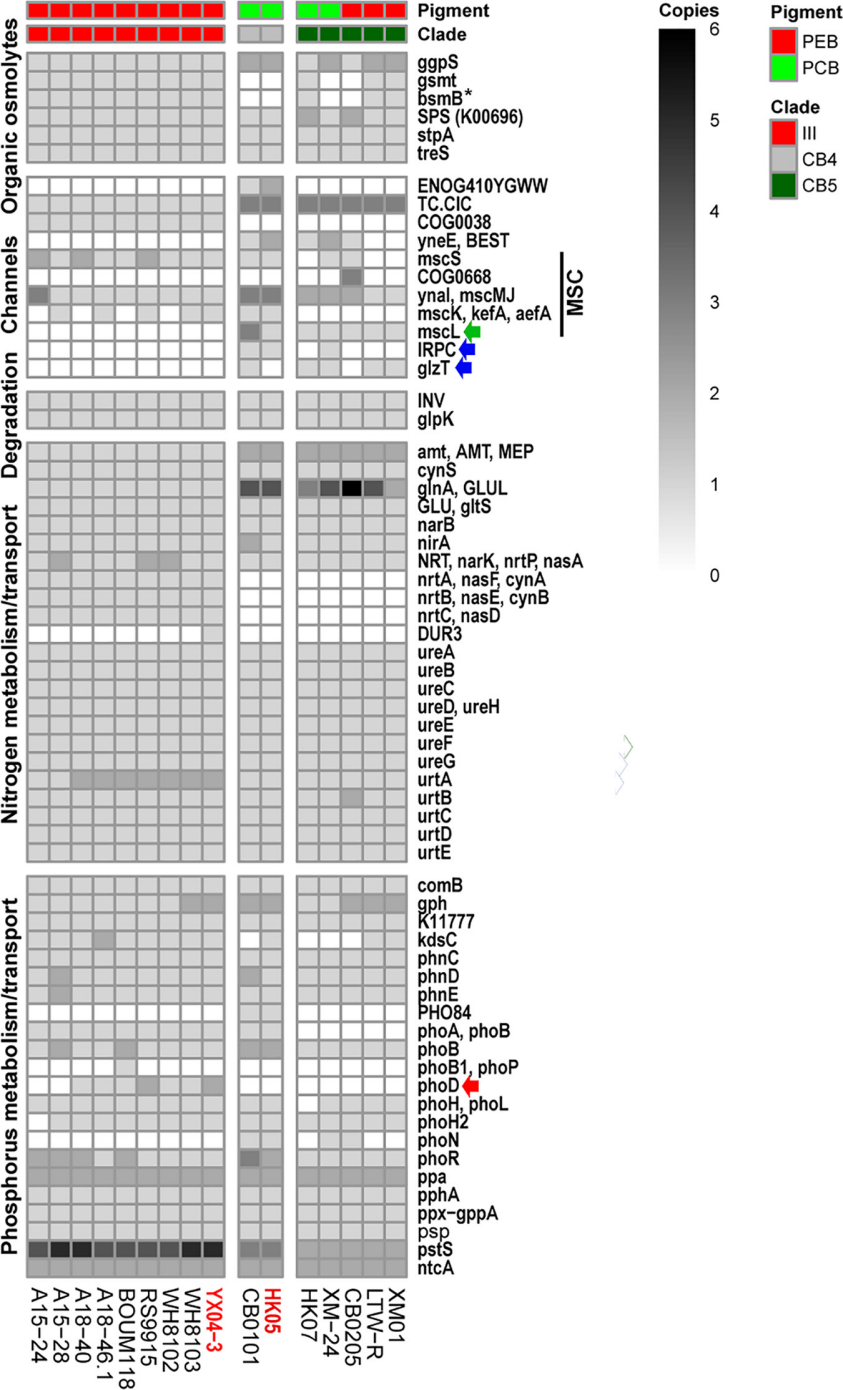
Genome comparison of *Synechococcus* strains of clades III, CB4 and CB5. Key genes that are involved in organic osmolytes biosynthesis, channel protein biosynthesis and degradation, nitrogen metabolism and transport, and phosphorus metabolism and transport were shown. *bsmB gene was previously identified as *sdmt* gene using the eggNOG-Mapper v2. MSC, mechanosensitive channel.

10.1128/msystems.01106-22.3 FIG S3 The growth and photosynthesis properties of *Synechococcus* strains YX04-3 and HK05 at salinities of 13 ppt and 32 ppt. (A) The growth of YX04-3. (B) The growth of HK05. (C) The growth of HK05 at 13 ppt and 32 ppt, after 4 days of acclimation (for details, see Fig. 2A). (D) OD640 nm/OD440 nm values of HK05. E, The maximal PSII photochemical efficiency (Fv/Fm) of HK05. Download FIG S3, DOCX file, 0.2 MB. Copyright © 2023 Xia et al. 2023 Xia et al. https://creativecommons.org/licenses/by/4.0/ This content is distributed under the terms of the Creative Commons Attribution 4.0 International license.

We analyzed the genome of YX04-3 and HK05, and found that YX04-3 had a genome size of 2.46 Mb, which is smaller than that of HK05 (2.53 Mb) (Data set S2). In addition, the GC content of YX04-3 (59.3%) was lower than HK05 (64.0%). YX04-3 contains pigment genes *cpcBA* (phycocyanin), *cpeBA* (phycoerythrin I) and *mpeBA* (phycoerythrin II), while HK05 only has *cpcBA*, suggesting that YX04-3 is a PUB-containing *Synechococcus* and HK05 is a PCB-rich *Synechococcus* (37) (Data set S2). This result is consistent with previous studies that PCB-rich *Synechococcus* is mainly distributed in estuarine waters (14, 34, 42).

**FIG 3:**
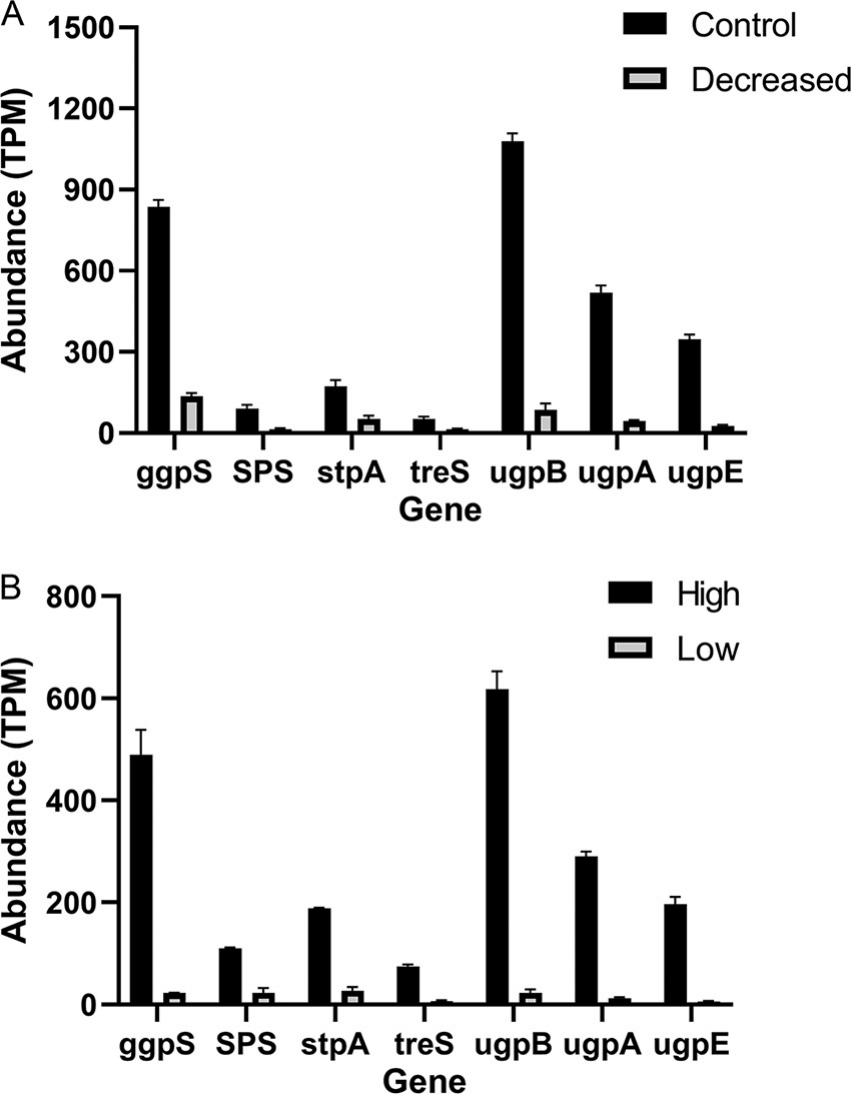
The abundance of organic osmotic genes and sugar transporter genes in the different salinities. (A) Change of organic osmotic genes and sugar transporter genes abundance when salinity suddenly dropped from 32 ppt (Control) to 13 ppt (D). (B), The abundance of organic osmotic genes and sugar transporter genes in the low salinity (13 ppt) and high salinity (32 ppt) conditions after acclimation. For more details about the experimental setup, see Fig. 4A.

The genome profiles of YX04-3 and HK05, as well as other strains of clade III and CB4/CB5, were compared (Fig. 2 and Data set S2). To adapt to high salinity environments, *Synechococcus* strains commonly utilize sucrose, trehalose, glucosylglycerol, and glycine betaine as organic osmolytes (43). Previous studies have suggested that there is a rough correlation between the salt tolerance of Cyanobacteria strain and organic osmolytes used to balance the osmotic potential (44, 45). Cyanobacteria in freshwater with low salt tolerance usually use sucrose and trehalose, while those in marine environments with moderate salt tolerance often use glucosylglycerol. Glycine betaine is a major organic osmolyte of halophilic Cyanobacteria in extremely saline environments. Our genomic analysis showed that the *SPS*, *ggpS*, *treS* and *stpA* genes, which are involved in synthesizing sucrose, glucosylglycerol and trehalose, are present in the genomes of clade III and CB4 strains, whereas the *bsmB* and *gsmt* genes which are related to the synthesis of glycine betaine are detected in genomes of clade III but not in genomes of CB4 strains (Fig. 2). However, all CB4 strains have 2 copies of *ggpS* genes. In addition, the transcript abundance of the *ggpS* gene of CB4 strain HK05 was markedly higher than other compatible solute genes in high salinity conditions (Fig. 3). These results suggest that CB4 euryhaline *Synechococcus* may use glucosylglycerol as the main organic osmolyte, similar to many other freshwater Cyanobacteria (46, 47), while clade III strictly marine *Synechococcus* may use glycine betaine to cope with hyperosmotic stress (48, 49). The *bsmB* and *gsmt* genes were mainly distributed in strictly marine *Synechococcus* clades, supporting a previous observation that glycine betaine encoding genes were only detected at salinities > 16 ppt in the Baltic Sea (13). *Synechococcus* utilize different osmotic compatible solutes to cope with high salinity stress (13, 18), which may be a reason for their salinity preferences and distributions in euryhaline environments (25).

To cope with a reduction in salinity, euryhaline microorganisms might achieve a balance in osmotic pressure between the cell interior and exterior by either releasing or degrading intracellular enriched osmotic compatible solutes (44). For instance, *Synechocystis* immediately release a large amount of glucosylglycerol to the ambient environment to cope with the sudden hypoosmotic shock. It has been suggested that mechanosensitive channels (Msc) are involved in the rapid release of dissolved substances (44, 50). Among them, MscL has been demonstrated to be essential for surviving hypoosmotic shock. In comparison, we indeed found more *msc* genes (*mscS*, *ynaI*, *mscK*, *mscL*) in the genomes of euryhaline clade CB4 than in clade III. Specifically, the *mscL* gene, which is involved in the synthesis of MscL, was not detected in the genome of all clade III strains (Fig. 2). This explained why the clade III strain YX04-3 did not grow well when it was transferred from a high salinity environment to a low salinity environment. In addition, the *glzT* and *IRPC* genes, which are presented in most CB4/5 strains (Fig. 2), might also be involved in the low salinity adaptation (18). We further checked the key genes which are involved in degrading intracellular enriched osmotic compatible solutes in the *Synechococcus* genomes. Those genes include the *gghA* (glucosylglycerol hydrolase A), *glpK* (glycerol kinase) and *Inv* (sucrose invertase). The *Inv* gene was detected in nearly all *Synechococcus* genomes, while the *gghA* gene which is responsible for the depletion of the intracellular osmotic compatible solute glucosylglycerol was absent in all *Synechococcus* genomes, although the *ggpS* and *glpK* genes which are often co-located with *gghA* were found in *Synechococcus* genomes (Fig. S4) (44, 51). Together, these results might suggest that clade III has a low abundance in brackish water due to the lack of the key mechanosensitive channel gene *mscL*. Euryhaline *Synechococcus* strains adapt to a decrease of salinity by releasing compatible solute glucosylglycerol instead of degrading it.

10.1128/msystems.01106-22.4 FIG S4 Distribution of the glucosylglycerol (*GG*) synthesis (*ggpS*) and degradation (*gghA*) genes, and the mechanosensitive channel (mcsS) gene in the HK05, YX04-3 and PCC6803 genomes. *ggpS*, GG-phosphate synthase; *glpK*, glycerol kinase; *gghA*, GG hydrolase A; *glpD*, glycerolphosphate dehydrogenase. Download FIG S4, DOCX file, 0.2 MB. Copyright © 2023 Xia et al. 2023 Xia et al. https://creativecommons.org/licenses/by/4.0/ This content is distributed under the terms of the Creative Commons Attribution 4.0 International license.

We also compared the genome sequences of clade III and CB4 strains to investigate probable reasons for their widely different spatial distributions. With regard to nitrogen metabolism, these 2 clades could both utilize ammonia, nitrate, and nitrite as inorganic nitrogen sources, since the *glnA*, *narB*, and *nirA* genes were all present in their genomes (Fig. 2). CB4 strains might have a higher ability to uptake and assimilate ammonia than clade III strains because they have 2 copies of the *amt* gene (which is involved in ammonia transport), and 4 copies of the *glnA*/*GLUL* genes (which are known to be involved in ammonia assimilation). In contrast, clade III strains might have a higher ability to uptake nitrite/nitrate, as they have *nrtABC* transporter genes which are involved in nitrite/nitrate import. CB4 strains have more genes involved in ammonia transport, whereas clade III strains have more genes involved in nitrate/nitrite transport, corresponding to the distribution of different nitrogen sources in estuarine waters and open ocean waters. Finally, both clade III and CB4 could utilize urea as the source of nitrogen, because urea transporter genes (*urt*) and urease genes (*ure*) were present in their genomes.

*Synechococcus* clade III has been reported well adapted to phosphorus-depleted waters (3, 38). A previous study suggested clade III strain WH8102 might adapt to phosphorus depletion by 2 strategies, the first being *PhoB*-dependent induction of high-affinity PO_4_ transporters, and the second being *PtrA*-dependent (a cAMP receptor protein gene that shows homology to *NtcA*) induction of phosphatases for scavenging organic phosphorus (52). However, *phoB* gene and *ptrA* (was classified as *ntcA* gene in the present study [53]) were detected in all genomes of clade III and CB4 strains, as well as other *Synechococcus* strains (Fig. 2). Thus, these strategies might not explain why clade III can adapt to phosphorus-depleted waters. Recently, it has been suggested that phosphatase genes may also play essential roles for Cyanobacteria in the adaption of P-depleted environments (39). The *ppa* and *ppx* which encode enzymes involved in the hydrolysis of inorganic phosphate polymers and releasing the terminal orthophosphate group from linear polyphosphates, respectively, were found in all genomes. Alkaline phosphatase genes (APase) *phoA*, *phoD*, and *phoX* are commonly found in cyanobacterial strains (54). However, only the *phoA* gene was detected in all clade III and CB4 genomes. The *phoD* gene was only distributed in clade III strains (YX04-3 has 2 copies of *phoD* gene) and the *phoX* gene was not detected (Fig. 2). In addition, the *pstS* gene which is a key gene involved in the transportation of phosphate has higher abundant in clade III (4-5 copies) compared with CB4 and CB5 strains. Therefore, the adaption of clade III to phosphorus-depleted waters might depend on its particularly high number of phosphatase and transporter genes that allow it to utilize diverse sources of organic phosphorus.

### Strategies of clade CB4 strain HK05 to adapt to sudden drop in salinity.

**FIG 4:**
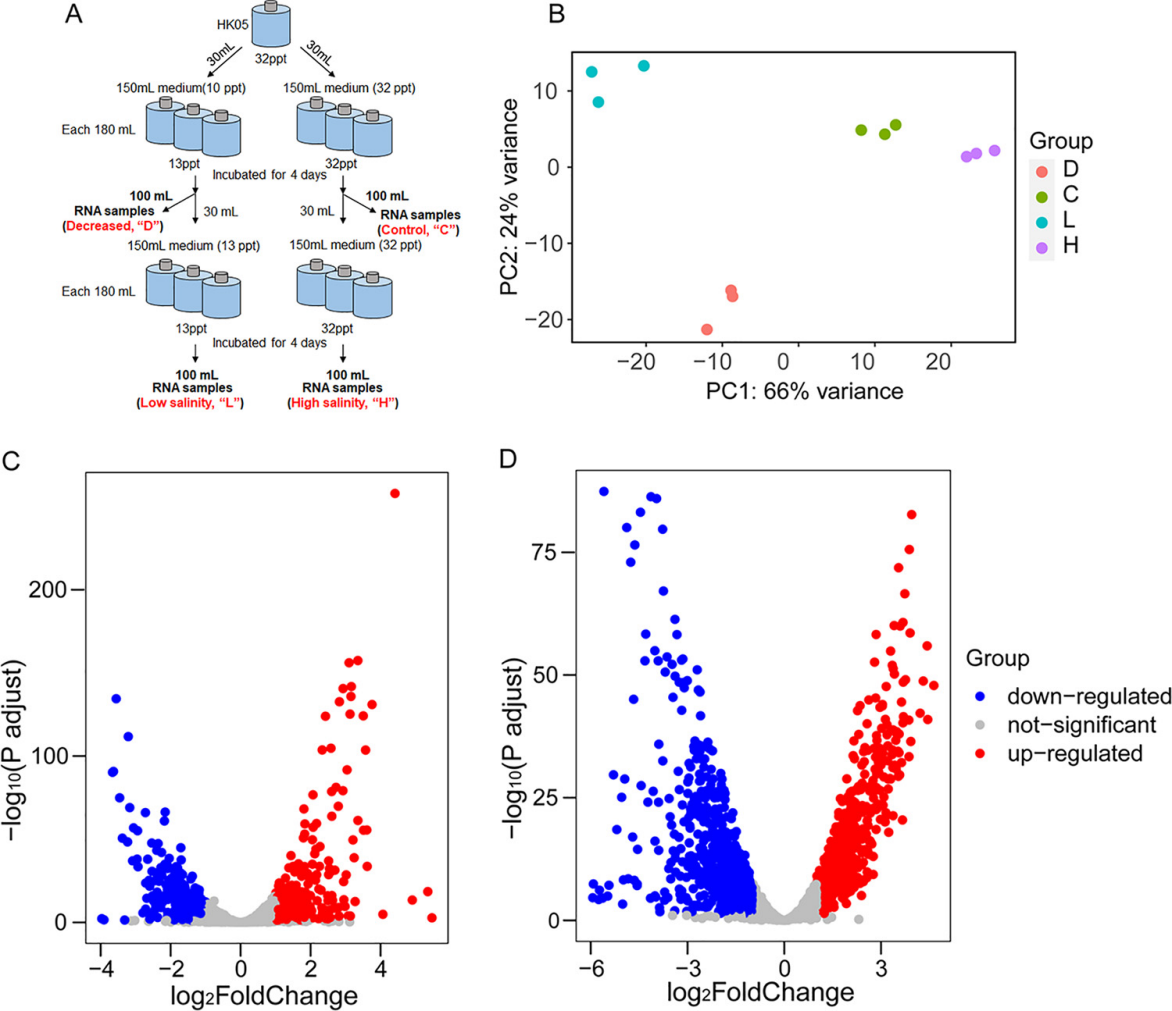
Transcriptome analysis of HK05 in different salinity conditions. (A) Flowchart of the experimental setup. (B), Principal-component analysis (PCA) of the transcriptomic profiles of HK05 at different salinities. Samples of the “D” and “L” groups (represented by the red and blue dots, respectively), had been kept at a salinity of 13 ppt, whereas those of the “C” and “H” groups (represented by the green and purple dots, respectively), had been kept at 32 ppt. (C) Volcano plot of differentially expressed genes (DEG) in HK05 under D (decreased salinity) and C (control) conditions. Blue and red dots indicate genes significantly reduced or enhanced in the D condition compared with the C condition. (D) Volcano plot of differentially expressed genes (DEG) in HK05 under L (low salinity) and H (high salinity) conditions. Blue and red dots indicate genes significantly reduced or enhanced in the L condition compared with the H condition.

**FIG 5:**
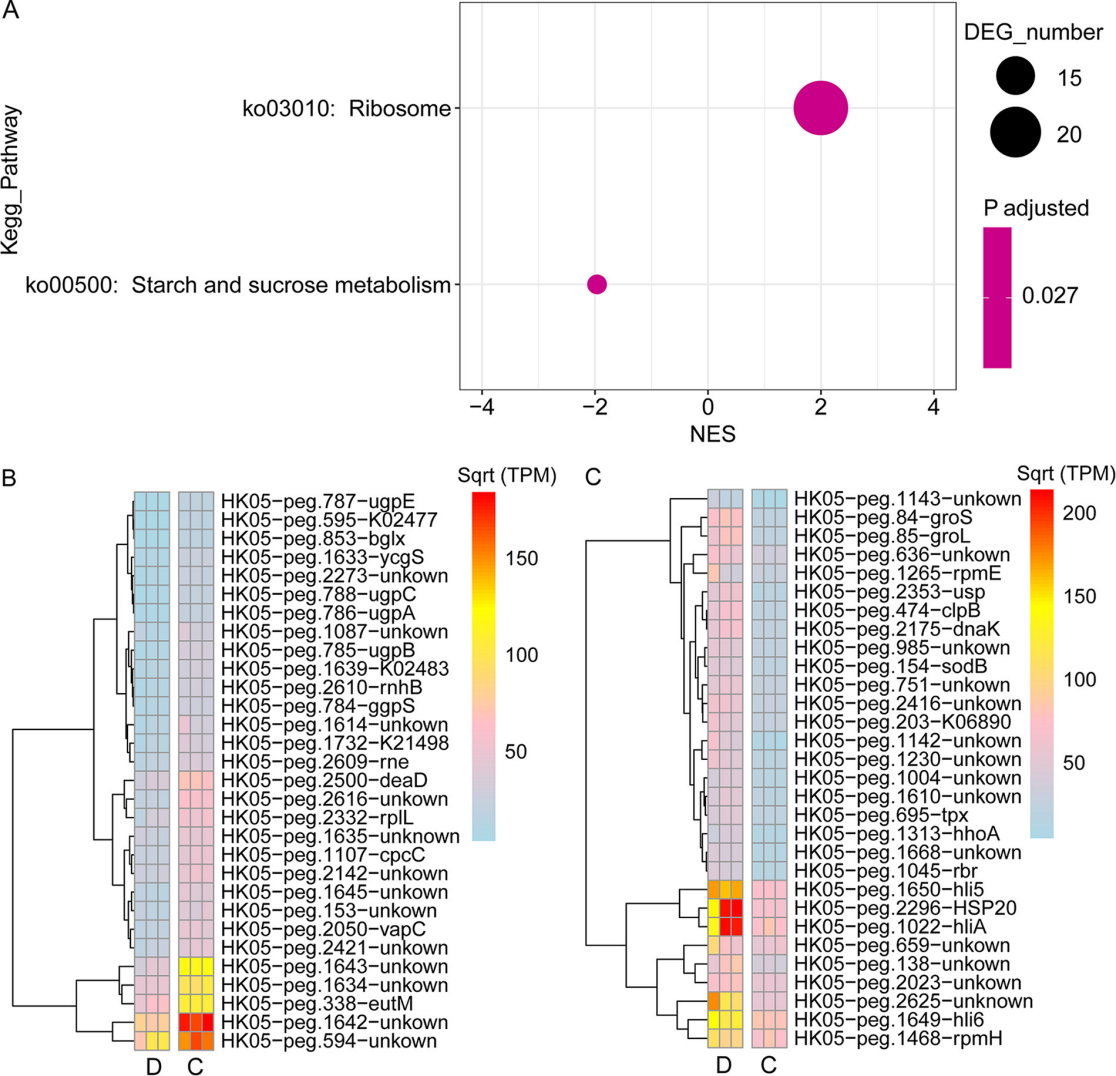
Transcriptome analysis of HK05 in response to the decrease of salinity. (A) GSEA analysis of KEGG pathways that were significantly downregulated or upregulated in the D (decreased salinity) condition compared with the C (control) condition. Positive or negative normalized enrichment scores (NES) indicate that the pathways were positively or negatively enriched in the D condition. (B) Heatmaps showing the abundance of the top 30 downregulated genes (evaluated using the A-C index defined by this study) in the D condition. (C) Heatmaps showing the abundance of the top 30 upregulated genes (evaluated using the A-C index defined by this study) in the D condition.

A previous study showed that the transcriptome of *Synechococcus* sp. 7002, a euryhaline cyanobacterium, grown at low salinity (3 mM NaCl and 0.08 mM KCl) had relatively minor changes relative to cells grown under standard conditions (300 mM NaCl and 8 mM KCl) (55). However, we observed that the transcriptome of HK05 could be strongly affected by variations in salinity (Fig. 4). PCA analysis showed that samples were separated into 3 main distinct clusters (Fig. 4B). Samples from “C” and “H” treatments were clustered together (salinity: 32 ppt). However, samples of the “D” and “L” treatments were separated into 2 clusters, even though they were incubated in the same salinity (13 ppt). This might be because the cells in the “D” group had to cope with the sudden drop in salinity. Indeed, in the “D” group (when HK05 was transferred from high salinity (32 ppt) to low salinity (13 ppt) medium), 700 genes were differentially expressed (|Log_2_[Fold change]|> 1, *P*adj < 0.05) (Fig. 4C). Among them, 359 genes were upregulated and 341 genes were downregulated. KEGG pathway significantly enriched for the upregulated transcripts was Ribosome (ko03010, hereafter not include rRNA genes), and the pathway significantly enriched for the downregulated transcripts was the starch and sucrose metabolism (Fig. 5A). Upregulation of ribosomal proteins is related to higher growth rate of HK05 in low salinity conditions, supporting the conclusion of previous studies that ribosomal proteins were essential for cell growth and adaptation to environmental changes (56–58). Function enrichment analysis with KEGG BRITE terms further showed that besides Ribosome (ko03011), Exosome (ko04147) and Chaperones and folding catalysts (ko03110) were enriched with decreasing of salinity (Fig. S5A). KEGG BRITE significantly enriched for the downregulated transcripts was Transporters (ko02000) (Fig. S5A).

10.1128/msystems.01106-22.5 FIG S5 Comparison of transcriptome profiles of HK05 in different salinity conditions. (A) GSEA analysis of KEGG BRITEs that were significantly down-regulated or up-regulated in the D (decreased salinity) condition compared with the C (control) condition. (B) GSEA analysis of KEGG BRITEs that were significantly down-regulated or up-regulated in the L (low salinity) condition compared with the H (high salinity) condition. Positive or negative normalized enrichment scores (NES) indicate that KEGG BRITEs were positively or negatively enriched in the D/L condition. Download FIG S5, DOCX file, 0.2 MB. Copyright © 2023 Xia et al. 2023 Xia et al. https://creativecommons.org/licenses/by/4.0/ This content is distributed under the terms of the Creative Commons Attribution 4.0 International license.

In the “D” group, HK05 also exhibited a reduced capacity for the biosynthesis of organic osmotic compounds, corresponding to the decrease of salinity, with a lower relative abundance of the *ggpS* (Log_2_[Fold change] = −2.17), *SPS* (−2.26), *stpA* (−1.19) and *treS* (−1.33) genes (Fig. 3A). The *ggpS* transcripts decreased strongly when HK05 was transferred from the salinity of 32 ppt to 13 ppt, supporting the point that glucosylglycerol is likely to be a major osmotic compound of the euryhaline PCB-rich *Synechococcus*. A similar result has been obtained by Ludwig et al., who showed that transcripts for *ggpS* and *stpA* of *Synechococcus* sp. 7002 were strongly downregulated at low salinity conditions (55). We further observed that transcripts of genes that are involved in transporting sugars from ambient seawaters into cells were strongly and significantly reduced in response to the decrease of salinity (e.g., *ugpA*, *ugpB* and *upgE*) (Fig. 3A and 5B). These results suggest that one major strategy by euryhaline *Synechococcus* to cope with a sudden drop of salinity is sharply reducing the osmotic compounds biosynthesis and sugar transportation.

Genes that are known to participate in the stress response, including heat shock protein (HSP) genes *HSP20*, *groS*, *groL*, *dnaK* and high light-inducible protein genes *hliA*, *hli5* and *hli6*, were all significantly enhanced by the decrease in salinity (Fig. 5C). In addition to salt stress, transcripts of these heat shock protein genes are also found strongly induced by thermal stress, high light stress, UV and oxidative stress (59). On the other hand, it has been suggested that high light-inducible proteins encoded by the *hli* genes could be involved in the regulation of tetrapyrrole biosynthesis in response to the cellular demand of chlorophyll and play a critical role in photoprotection (60, 61). In *Synechocystis* (another picocyanobacteria), the expression of *hli* genes can be induced by high light stress, cold stress, and nutrient starvation (62, 63). Upregulating the transcripts of HSP genes and high light-inducible protein genes may play an essential role in protecting the photosystem of *Synechococcus* in volatile environments.

In general, to adapt to the sudden drop of salinity, euryhaline strain HK05 exports osmotic compounds via mechanosensitive channels and downregulates biosynthesis of organic osmotic compounds and transporters to maintain cellular osmotic homeostasis; while at the same time, it upregulates transcripts of ribosomes, heat shock protein genes, and high light-inducible protein genes to synthesize and accumulate proteins in response to stress (especially for protecting the photosystem).

### Transcriptomic profiles of clade CB4 strain HK05 at high and low salinity conditions (after high or low salinity acclimation).

**FIG 6:**
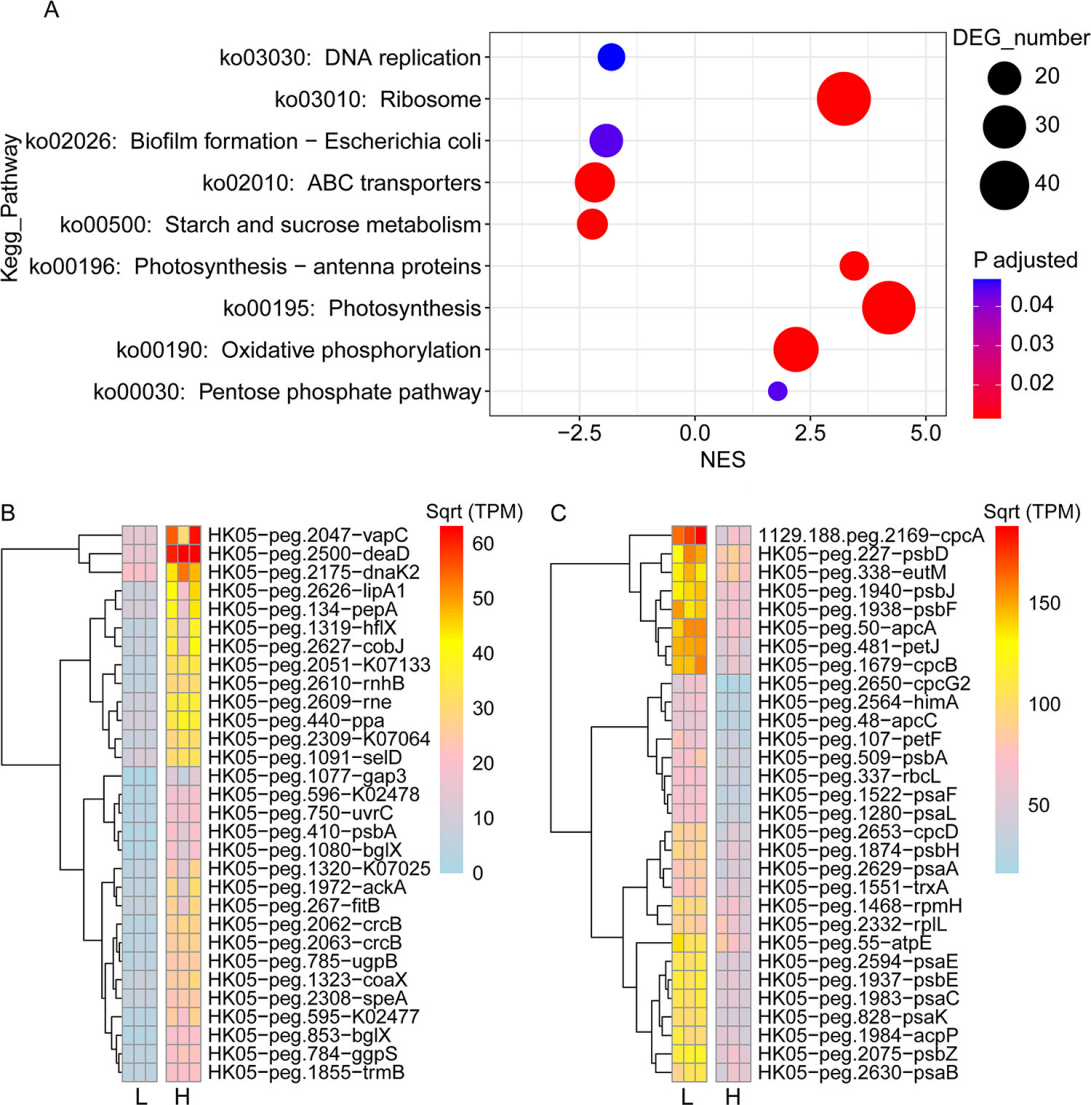
Comparison of transcriptome profiles of HK05 in the low and high salinity conditions. (A) GSEA analysis of KEGG pathways that were significantly downregulated or upregulated in the L (low salinity) condition compared with the H (high) condition. Positive or negative normalized enrichment scores (NES) indicate that the pathways were positively or negatively enriched in the L condition. (B) Heatmaps showing the abundance of the top 30 downregulated genes (evaluated using the A-C index defined by this study) in the L condition. (C) Heatmaps showing the abundance of the top 30 upregulated genes (evaluated using the A-C index defined by this study) in the L condition.

When comparing the “H” (salinity 32 ppt) and “L” (salinity 13 ppt) groups, 599 genes were upregulated and 661 genes were downregulated in the low salinity condition (Fig. 4D), corresponding to 4 significantly upregulated KEGG pathways and 5 significantly downregulated pathways (Fig. 6A). Pathways involved in photosynthesis, photosynthesis-antenna proteins, oxidative phosphorylation, pentose phosphate pathway, and ribosome were significantly positively regulated, whereas those related to DNA replication, biofilm formation, ABC transporters, and starch and sucrose metabolism were negatively regulated (Fig. 6A). KEGG BRITEs significantly enriched for the downregulated transcripts were Transporters (ko02000) and DNA repair/recombination proteins (ko03400), while those enriched for the upregulated transcripts were Ribosome (ko03011) and Photosynthesis proteins (ko00194) (Fig. S5B).

**FIG 7:**
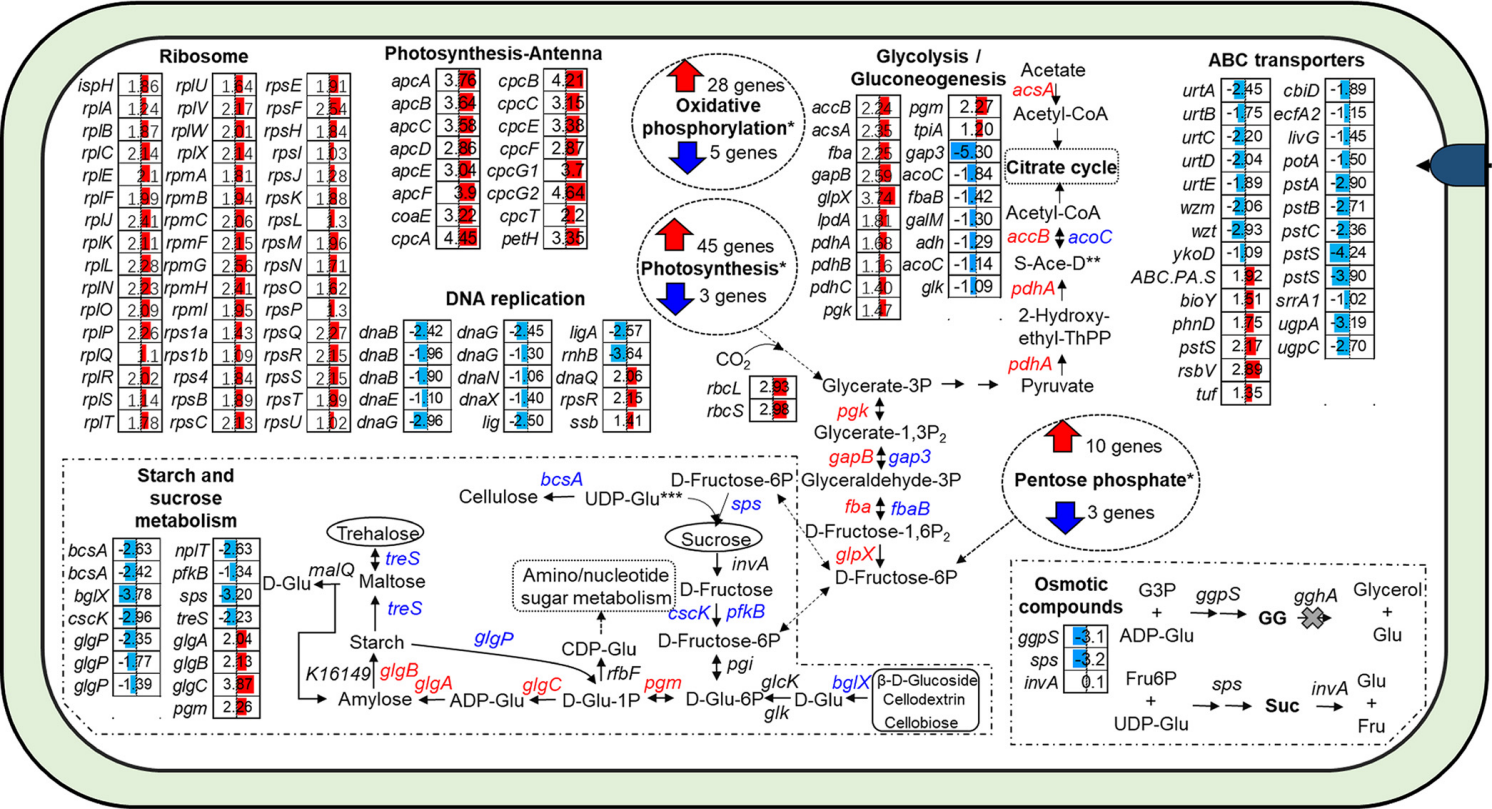
The genes and pathways upregulated or downregulated in the “L” condition compared with the “H” condition. The heatmaps demonstrate the Log_2_(Fold change) values of the DEGs. Cells in the “L” condition were pre-incubated in low salinity (13 ppt) medium and then kept at the same salinity level, whereas those in the “H” group were pre-incubated in high salinity (32 ppt) medium and kept at this same salinity (Fig. 2A). *, More details are shown in Data set S3; S-Ace-D, S-Acetyl-Dihydrolipoamide-E; Glu, Glucose.

This study, together with 2 previous studies (55, 64), showed that freshwater or euryhaline *Synechococcus* grew faster in low salinity conditions than in high salinity conditions. Transcriptome analyses suggest that high growth rate of HK05 in the low salinity condition could be attributed to 2 reasons: One is that, to adapt to low osmotic pressure, HK05 reduced the biosynthesis of osmotic compounds (*ggpS* gene in Fig. 6B), which in turn allowed more carbon to be used for growth. The other reason is that photosynthesis of HK05 was strongly enhanced in the low salinity condition, as evidenced by the high expression of genes governing the biosynthesis of antenna proteins, photosynthesis-related proteins, carbon fixation genes and ribosomes (Fig. 6C and 7). For example, *rbcS* and *rbcL* (the genes for Rubisco subunits) were enhanced by 7.63 and 7.90 folds (log_2_[Fold change] = 2.93 and 2.98) in the low salinity condition, respectively (Fig. 7). We noted that DNA replication pathway was downregulated in the low salinity condition where HK05 had a higher growth rate. However, the underlying reason for this observation remains unknown.

10.1128/msystems.01106-22.8 DATA SET S3 Enriched KEGG pathways in the high salinity treatments. Download Data Set S3, XLSX file, 0.04 MB. Copyright © 2023 Xia et al. 2023 Xia et al. https://creativecommons.org/licenses/by/4.0/ This content is distributed under the terms of the Creative Commons Attribution 4.0 International license.

In the low salinity condition, although the pathway of starch and sucrose metabolism was negatively regulated, we found that genes related to the biosynthesis of starch (e.g., *glgB*, *glgA*, *glgC*, and *pgm*) were strongly enhanced (Fig. 7). This suggests that reducing the salinity can enhance the production and accumulation of starch in euryhaline *Synechococcus*, as has been reported for various other marine microalgae (65).

### Conclusions.

Here, we showed that in subtropical estuarine waters, SC5.2 ecotypes of *Synechococcus* are often more abundant than SC5.1 ecotypes. In addition, despite their high phylogenetic diversity, SC5.2 *Synechococcus* in Pearl River estuary mainly belong to ecotype SC5.2-CB4. Unlike SC5.1 PEB-containing *Synechococcus*, SC5.2-CB4 PCB-rich *Synechococcus* utilize glucosylglycerol as a major osmotic compound. Our laboratory-based investigations revealed that SC5.2-CB4 strain HK05, grew faster at low salinity than at high salinity, justifying its high abundance in estuarine waters. The higher growth rate at low salinity is due to the enhancement in photosynthesis, biosynthesis of antenna proteins and biosynthesis of ribosome. This *Synechococcus* strain can survive and grow in low salinity waters possibly because it has multiple mechanosensitive channels. Our next study will contextualize these findings with field-based investigations using the metagenomic and metatranscriptomic approaches. Our new findings show that SC5.2 *Synechococcus* strains are better able to cope with environmental fluctuation than SC5.1 clades. However, their distribution is largely confined to estuaries/brackish waters/freshwaters, and they are rare in coastal and oceanic waters. Further studies are now under way to reveal the mechanisms underlying this phenomenon.

## MATERIALS AND METHODS

### Sample collection and environmental analysis.

Samples were collected from 28 stations in the Pearl River estuary in July 2018 when the annual peak abundance of *Synechococcus* occurs (14). At each station, the surface water was collected using a conductivity-temperature-depth rosette system (CTD, Sea-Bird Electronics). Approximately 0.5 L of water from each station was pre-filtered through a 3 μm polycarbonate (PC, Millipore Corporation) membrane and then filtered onto a 0.2 μm PC membrane. The membranes were stored in a −80°C freezer until their analysis on land. The salinity and temperature of each station were measured by the CTD. PAR which is often correlated with water turbidity was retrieved from the NOAA database (https://coastwatch.pfeg.noaa.gov) (66, 67). Nutrients including PO_4_^3+^, Si, NO_3_-, NO_2_-, and NH_4_^+^ were measured with a Lachat 8500 nutrient analyzer.

### Flow Cytometry analysis of PCB-rich and PEB-containing *Synechococcus* abundance.

For counting *Synechococcus* abundance, 1.8 mL surface water from each sampling station was collected and fixed with 50 μL seawater-buffered paraformaldehyde (0.5%, final concentration) in the dark for 15 min, before being flash-frozen with liquid nitrogen. The cell abundance of the PCB-rich and PEB-containing *Synechococcus* were enumerated using a Becton–Dickinson FACSCalibur cytometer equipped with dual lasers at 488 nm and 635 nm, following the method described previously (14).

### Genomic DNA extraction, PCR of the *rpoC1* gene, and sequence analysis.

Genomic DNA was extracted from the 0.2 μm membranes using the PureLink Genomic DNA Extraction kit (Invitrogen). Amplification of the *rpoC1* gene sequences was performed with the rpoC1-39F and rpoC1-462R primer pair, as previously described (19). The PCR products were gel-purified using the Qiaquick gel purification kit (Invitrogen) and sequenced using an Illumina HiSeq 2500 platform (Novogene Company).

Analysis of the *rpoC1* gene sequences was conducted using the Mothur software package (http://www.mothur.org) according to a protocol we described previously (7). In brief, the raw reads were assembled using the *make.contigs* command, and then chimera sequences were identified using the *chimera.vsearch* command and removed from the data sets. Sequences with a length shorter than 300 nt or longer than 500 nt, were also removed. We then randomly subsampled 50,000 sequences from each sample using the *sub.sample* command to rarefy all the samples to the same sequencing depth. After these processing steps, sequence reads were aligned and classified by conducting a local BLAST against a reference FASTA file containing a set of *rpoC1* gene sequences (Data set S1, updated from Xia et al. [7]) using the *classify.seqs* command with 80% confidence in bootstrap values. Clades of *Synechococcus* were determined according to previous studies (5, 36). The relationships among the *Synechococcus* clades were characterized through Spearman correlation by using the cor function of R (68). Finally, high-quality sequences were clustered into operational taxonomic units (OTUs) at a 97% similarity level. The Bray-Curtis similarity matrix was built to carry out the non-metric multidimensional scaling (NMDS) analysis among the *Synechococcus* assemblage compositions in different samples using the R package vegan (69). The Mantel test was also performed using the R package vegan.

10.1128/msystems.01106-22.6 DATA SET S1 The *rpoC1* gene reference sequences used for classifying *rpoC1* reads obtained by Illumina HiSeq sequencing. Download Data Set S1, TXT file, 0.3 MB. Copyright © 2023 Xia et al. 2023 Xia et al. https://creativecommons.org/licenses/by/4.0/ This content is distributed under the terms of the Creative Commons Attribution 4.0 International license.

### Phylogenetic analysis of *Synechococcus* genomes.

For the phylogenetic analysis, 120 high-quality genome sequences of *Synechococcus* strains were downloaded from the NCBI database, including strains from freshwater systems, estuaries, brackish waters, and marine waters (Data set S2). The genomes were analyzed by using CheckM to obtain 43 concatenated phylogenomic marker genes alignments (70). The model test function in MEGA6 software package was then used for selecting the best model for the phylogenetic tree construction (71). A maximum likelihood (ML) phylogenetic tree was constructed using MEGA6 (71) based on JTT+G+I model. Bootstrapping was performed with 100 replications. For the *rpoC1* phylogenetic tree, sequences were extracted from the *Synechococcus* genomes and aligned using MEGA6, and then an ML tree was constructed using the GTR model.

### Isolation of *Synechococcus* strains and testing their responses to salinity variations.

*Synechococcus* strain HK05, which is affiliated with SC5.2-CB4, was isolated from the Pearl River estuary following the method described in our previous study (18), with slight modifications. In brief, water samples from the Pearl River estuary were filtered through a 1.2 μm PC membrane (Millipore Corporation) to remove large organisms. Then, 1 mL samples of filtered water were added to 3 mL modified f/2 medium (without Na_2_SiO_3_·9H_2_O but containing 100 μM NH_4_Cl), which was further diluted 5 times with seawater (0.2 μm PC membrane prefiltered). Cycloheximide (20 μg mL^−1^, final concentration), was added to the samples to restrict the growth of picoeukaryotes. The samples were then incubated under an illumination of ~25 μmol quanta m^−2^ s^−1^ at 25°C until slight pink or green coloration was observed. Ten-fold serial dilutions were then performed to obtain monoclonal cultures. The *Synechococcus* strains obtained were further identified by amplification of the *rpoC1* gene (19). Cultures that had multiple *rpoC1* genes or multiple pigment signals in the Flow Cytometry analysis were further purified using the 10-fold serial dilutions method. Culture YX04-3 (affiliated with SC5.1 clade III), had previously been isolated from the South China Sea, and was a gift from Dr. Qiang Zhen at Xiamen University.

To study the salinity tolerance of *Synechococcus* strains, the growth rates of HK05 and YX04-3 in modified f/2 medium with a salinity of 13 ppt or 32 ppt, were evaluated. The cultures were grown in plant growth chambers under an illumination of ~25 μmol quanta m^−2^ s^−1^ at 25°C in a 12 h/12 h light-dark cycle. The optical density at 440 nm (OD440) of each culture was measured every day, following the method described by Xia et al. (19). The maximal PSII photochemical efficiency (Fv/Fm) of HK05 was measured at day 4 and day 8 using an AquaPen Ap-100C hand-held fluorometer (Photon Systems Instruments).

### Genome comparison of dominant *Synechococcus* strains.

The genomic DNA of HK05 was extracted using a DNA Extraction minikit (Invitrogen) following the manufacturer’s instructions, and sequenced using an Illumina Hiseq 2000 sequencing system (Novogene Company). The sequences obtained were assembled using Spades with k-mer 55 and 77 (72). To remove possible contigs from heterotrophic bacteria, those ≥2 kb were binned using MyCC (73). The quality of the obtained genomes was evaluated using CheckM (70), and the genome identified as being affiliated with Cyanobacteria was retained for subsequent analysis. The YX04-3 genome was obtained from the NCBI database (accession number: RHLE00000000.1).

To annotate the YX04-3 and HK05 genomes, the genome sequences of both were then submitted to the RAST Server for open reading frame (ORF) prediction (74). The predicted ORFs and amino acid sequences were annotated using eggNOG-mapper v2 (75), BlastKOALA (76) and the nr database with default settings (Data set S2).

We compared genomes of YX04-3 and HK05, as well as other strains of clade III and CB4. The distribution of genes involved in salinity adaption, channel proteins synthesis, and nitrogen and phosphorus metabolisms were compared.

### Transcriptomic analysis of HK05 under different salinities.

Some previous studies, as well as this study, observed that when salinity is lower than 15 to 16 ppt, the growth of strictly marine *Synechococcus* will be inhibited, while the growth of euryhaline strains will not be affected (13, 19). In addition, we observed that euryhaline *Synechococcus* was very abundant in estuarine waters where salinity ranges from 10 to 16 ppt, while strict marine *Synechococcus* dominated in marine waters with salinity higher than 30 ppt. To unveil how euryhaline *Synechococcus* (HK05) cope with low salinity stress, transcriptomic analysis of HK05 under salinities 13 ppt and 32 ppt was conducted. In brief, 30 mL exponential phase culture (32 ppt) was transferred to 150 mL fresh modified f/2 medium with a salinity of either 10 ppt (hereafter called the ‘Decreased’ or ‘D’ group salinity treatment: final salinity ~13 ppt), or 32 ppt (hereafter called the ‘Control’ or ‘C’ group: final salinity ~ 32 ppt). After incubation for 4 days, 100 mL of the culture was filtered onto 0.2 μm PC membranes. The membranes were collected and immersed in RNAlater (Ambion) for RNA extraction. Another 30 mL of the “D” or “C” group culture was respectively transferred to 150 mL fresh medium containing a salinity of 13 ppt (low salinity treatment, hereafter called the “L” group) or 32 ppt (high salinity treatment, hereafter called the “H” group). After incubation for a further 4 days, the cells were collected and immersed in RNAlater. All treatment groups were prepared and incubated in triplicate in 250 mL Nalgene PC bottles. RNA was extracted using TRIzol (Invitrogen). For transcriptomic sequencing, rRNA transcripts in total RNA samples were removed by using ALFA-SEQ rRNA depletion Kit (for Bacteria). Then samples were sent to Magigene Biotechnology Company (Guangzhou, China) for library construction and sequencing on an Illumina Novaseq6000 platform.

The raw sequence data were processed by Trimmomatic (v0.36) to remove low quality and adaptor sequences (77). Then clean sequences were mapped to rRNA gene sequences of HK05 and CB0101 (another clade CB4 strain) to remove the rRNA sequences by Bowtie2 (around 1.5% to 7.6% of total reads removed). Clean sequence reads were mapped to the HK05 ORFs using Salmon to assess the abundance of each ORF (78). The expression levels of each gene in the “C” and “D” or “H” and “L” groups were compared using DESeq2 (79). The differentially expressed genes (DEGs) with a Log_2_(Fold change) > 1 and adjusted *P*-value (*P*adj) < 0.05 were considered to be significant. Gene Set Enrichment Analysis (GSEA) was applied to analyze the KEGG pathway enrichment based on KEGG pathway maps and BRITE functional hierarchies by using the R package, clusterProfiler (80, 81). To identify the top abundant and changed transcripts, we calculated the A-C index, which we defined as being the Mean abundance (‘transcript per million (TPM)’) × Fold change. Top 30 upregulated and downregulated genes were selected for the heatmap analysis using the R package pheatmap.

### Data availability.

All sequences obtained from this study have been deposited in the National Center for Biotechnology Information (NCBI) Sequence Read Archive under accession number: PRJNA741056.
