# Supplementary figures and images for: Genomic and Transcriptomic Insights into Salinity Tolerance-Based Niche Differentiation of Synechococcus Clades in Estuarine and Coastal Waters
Source: mSystems. 2023 Jan 9;8(1):e01106-22. doi: 10.1128/msystems.01106-22 (PMC9948718; doi:10.1128/msystems.01106-22)

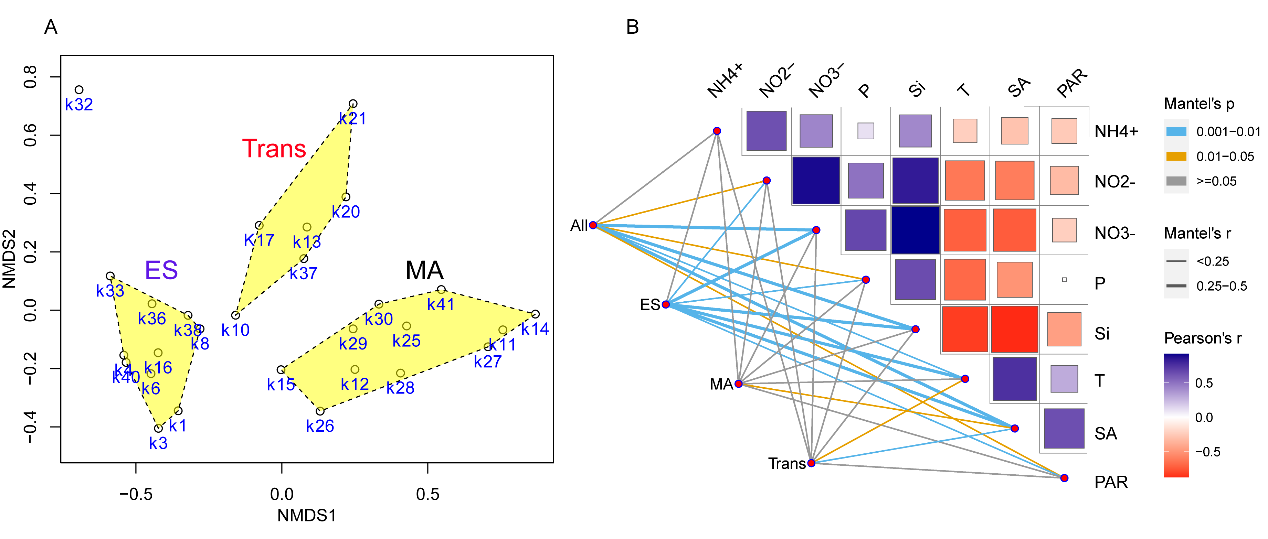

Supplement: FIG S1 [file msystems.01106-22-s0001.docx]

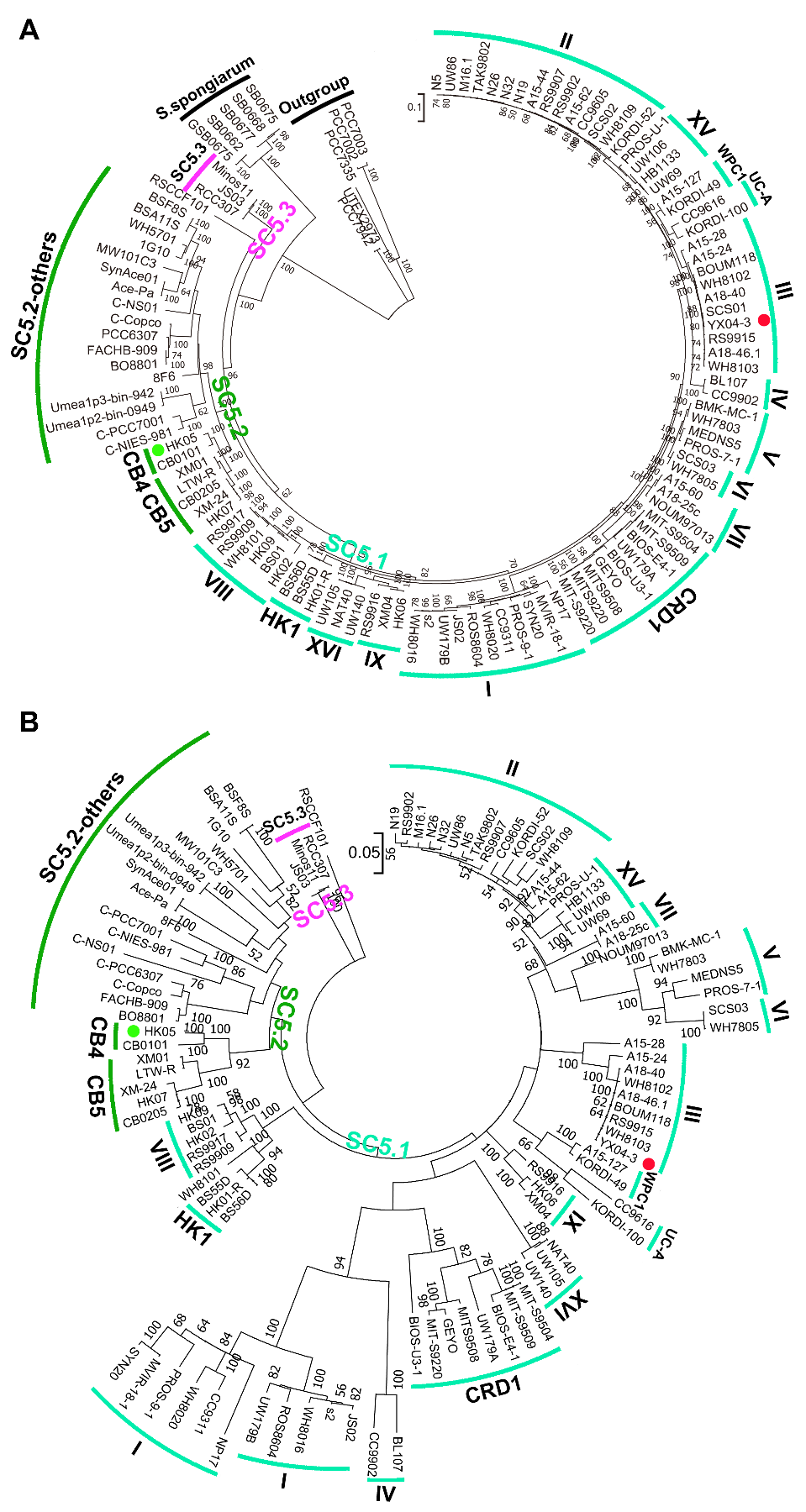

Supplement: FIG S2 [file msystems.01106-22-s0002.docx]

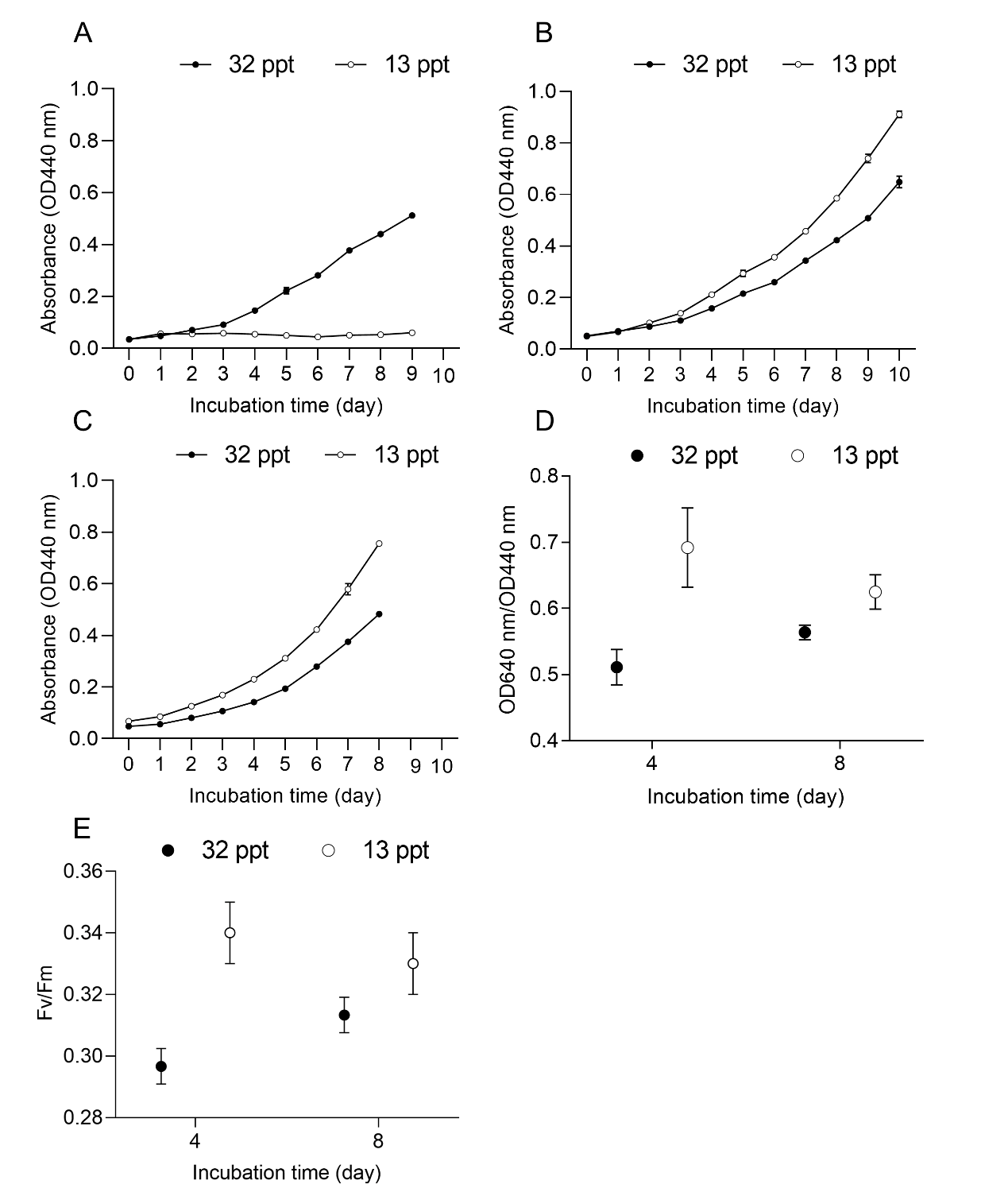

Supplement: FIG S3 [file msystems.01106-22-s0003.docx]

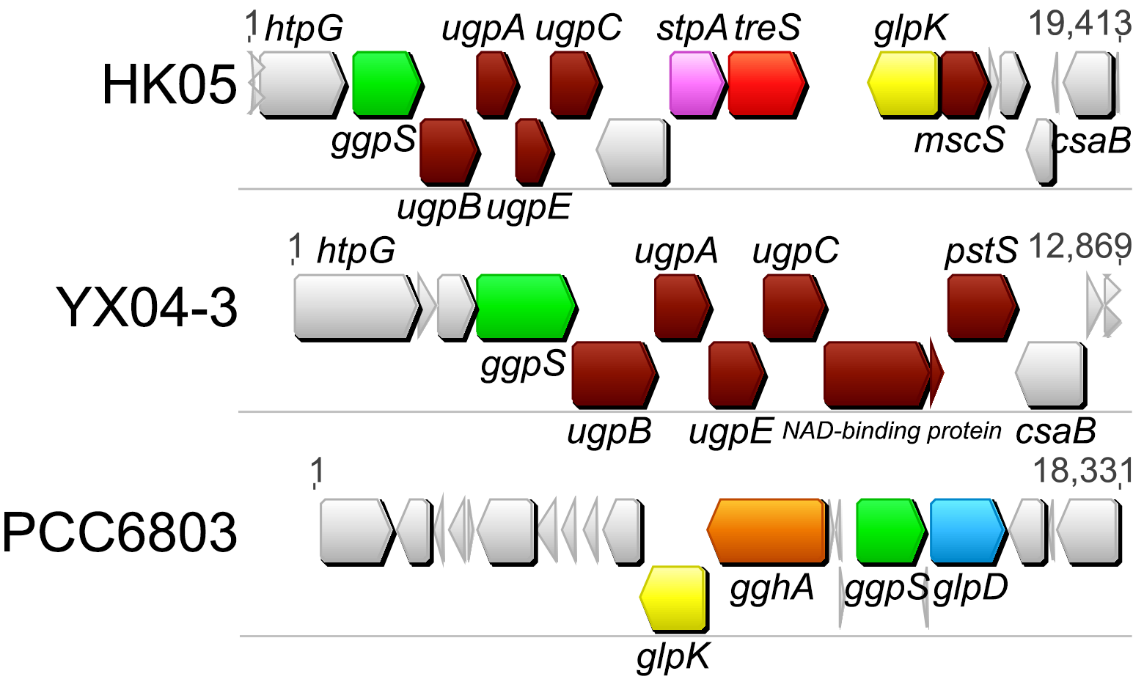

Supplement: FIG S4 [file msystems.01106-22-s0004.docx]

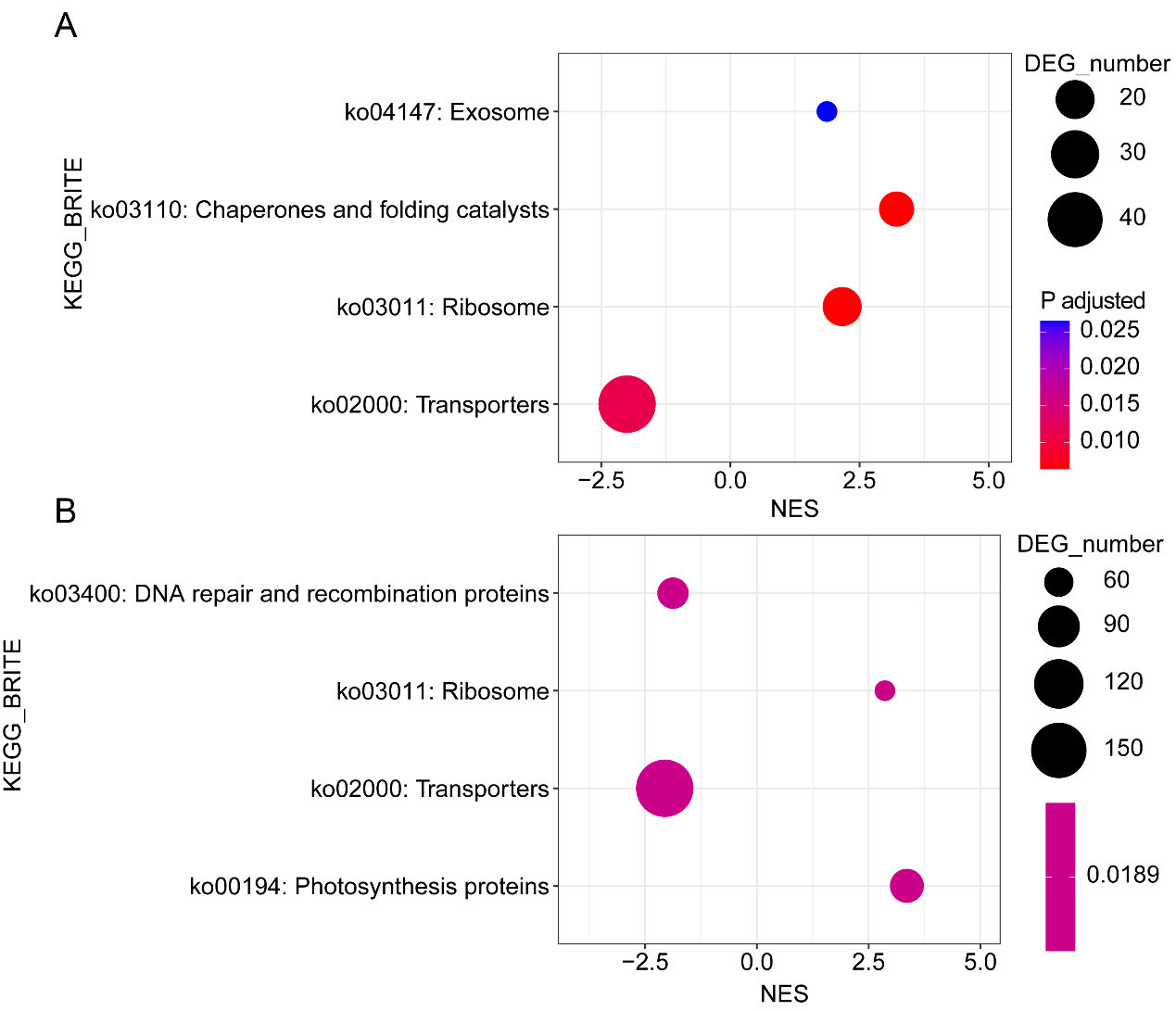

Supplement: FIG S5 [file msystems.01106-22-s0005.docx]
